# Spontaneous Entero-Cutaneous Fistula due to Femoral Hernia: A Case Report

**DOI:** 10.31729/jnma.v63i292.9269

**Published:** 2025-12-31

**Authors:** Rupesh Chaudhary, Nishant Thapa, Neeraj Gharti Magar, Saroj Pandit

**Affiliations:** 1 Kathmandu Medical College and Teaching Hospital, Sinamangal, Nepal; 2 Department of Surgery, Kathmandu Medical College and Teaching Hospital, Sinamangal, Nepal; 3 Emilio Aguinaldo College, Manila, Philippines

**Keywords:** *enterocutaneous fistula*, *femoral hernia*, *ileo-ileal anastomosis*, *lump in groin*

## Abstract

An enterocutaneous fistula is an aberrant connection between the intra-abdominal gastrointestinal tract and skin/wound. Spontaneous enterocutaneous fistula secondary to femoral hernia is extremely rare but may be life-threatening because of sepsis, malnutrition and stuid-electrolyte imbalance. We report an 83-year-old woman who presented with a short history of irreducible right groin swelling associated with feculent discharge. Contrast-enhanced computed tomography revealed a perforated distal ileal loop within a right femoral hernia, forming an enterocutaneous fistula. She underwent emergency laparotomy, segmental ileal resection with Meckel’s diverticulectomy, primary ileo-ileal anastomosis and hernia repair without mesh. Her postoperative recovery was uneventful and she remains well on follow-up. This case highlights the need to consider femoral Richter’s hernia with spontaneous enterocutaneous fistula in elderly women presenting with atypical groin discharge, and supports early imaging and definitive surgery to prevent morbidity.

## INTRODUCTION

Femoral hernia is a protrusion of abdominal contents through the femoral canal and has a high risk of strangulation (15-20%) among groin hernias.^1-3^ Incarceration may occur in up to 30% of cases, and elective repair is advised once diagnosed.^1,3^ Enterocutaneous fistula is an abnormal communication between the gastrointestinal tract and the skin; 70-90% arise postoperatively, most often after operations for malignancy, inflammatory bowel disease or adhesions.^5-7^ Spontaneous ECF due to femoral Richter’s hernia is exceptionally uncommon, with only a few cases reported.^4,9,10^

## CASE REPORT

**Figure 1:**
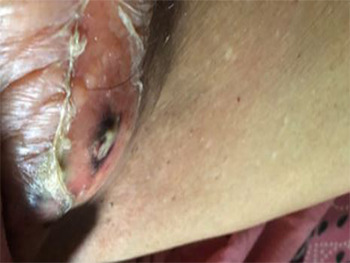
Swelling over the right groin.

An 83-year-old woman presented to the emergency department with a lump in the right groin for two weeks. Initially, the swelling was small and reducible but had gradually increased in size and became irreducible three days before presentation. Two days prior to admission she noticed spontaneous discharge of pus mixed with feculent material from the overlying skin and increasing local pain (Figure 1). There was no history of previous abdominal surgery, trauma, weight loss or altered bowel habit. On examination, she was afebrile and hemodynamically stable. The abdomen was soft and non-tender without features of obstruction or peritonitis. In the right inguinal region, just inferior and lateral to the inguinal ligament, there was a 3x4 cm tender, irreducible swelling with an overlying ulcer and feculent discharge, consistent with an enterocutaneous fistula. There was no generalized sepsis or skin cellulitis.

A working diagnosis of spontaneous enterocutaneous fistula secondary to strangulated femoral (Richter’s) hernia was made. Baseline laboratory investigations, including complete blood count, renal and liver function tests, and serum electrolytes, were within acceptable limits for emergency surgery.

**Figure 2:**
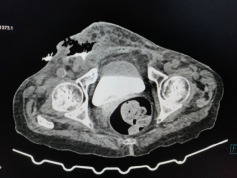
Contrast-enhanced computed tomography scan showing location of enterocutaneous fistula.

Contrast-enhanced computed tomography of the abdomen and pelvis demonstrated a segment of distal ileum herniating through the right femoral canal with mural thickening and discontinuity, associated with subcutaneous air and contrast extravasation in the right groin, confirming enterocutaneous fistula secondary to femoral hernia (Figure 2).

**Figure 3:**
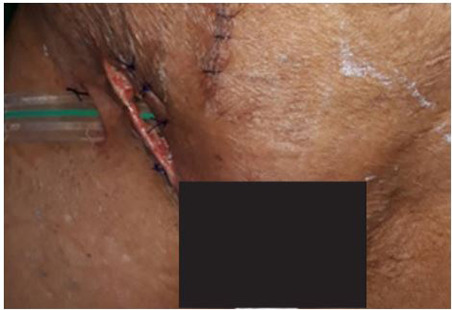
Second post-operative day showing clean wounds with drainage tube in-situ.

The patient underwent emergency midline laparotomy. Intra-operatively, approximately 1 cm of the antimesenteric border of the distal ileum, located 10 cm proximal to the ileocecal junction, was found incarcerated within the right femoral ring with evidence of strangulation and a small perforation communicating with the groin skin. There was localized contamination with foul-smelling enteric efstuent but no generalized peritonitis. A Meckel’s diverticulum was identified 20 cm proximal to the ileocecal junction. After thorough peritoneal lavage, the herniated segment and Meckel’s diverticulum were resected en bloc, and an end-to-end ileo-ileal anastomosis was performed. The femoral defect was repaired using primary tissue repair without mesh because of contamination. The groin wound and fistulous tract were debrided and irrigated. A diverting loop ileostomy was created to protect the anastomosis, and closed suction drains were placed (Figure 3).

Postoperatively, the patient was managed in the surgical ward with broad-spectrum intravenous antibiotics, fluid and electrolyte optimization, and early nutritional support. Bowel function returned on postoperative day 3. The drains were removed sequentially, and the groin wound showed progressive granulation without further fibula output. She was discharged on postoperative day 5 in Sable condition with a functioning ileostomy and clean wounds. At three-month follow-up, she remained asymptomatic with well-healed surgical scars, and reversal of the ileostomy was planned.

## DISCUSSION

Enterocutaneous fistula remains a challenging complication for surgeons because of its association with sepsis, fluid and electrolyte imbalance, malnutrition and prolonged hospital stay.^5-^^7^ The majority (70-90%) are postoperative and follow surgery for malignancy, inflammatory bowel disease or adhesions.^5-7^ Spontaneous ECF arising from a femoral Richter’s hernia is therefore extremely rare; only a few cases have been reported worldwide, including the case described by Chalise et al. in a similar age group.^9^ Our report adds to this limited literature and highlights important diagnostic and therapeutic considerations.

Femoral hernias account for 2-4% of all groin hernias and are more common in elderly women because of the wider female pelvis and relatively larger femoral canal.^1-3^ The tight femoral ring predisposes to Richter’s hernia, in which only part of the bowel circumference is incarcerated.^8^ As a result, luminal continuity may be preserved, so classical features of intestinal obstruction can be absent. Ongoing venous congestion and arterial compromise lead to localized necrosis and perforation of the entrapped segment, which may discharge externally and form an enterocutaneous fistula without generalized peritonitis, as in our patient.

This pathophysiology explains the diagnostic challenge: patients may present with a small, irreducible, tender groin swelling and minimal abdominal signs. Any overlying erythema, ulceration or atypical discharge should raise suspicion for impending or established fistulisation. In the present case, the absence of obstruction and peritonitis could have led to underestimation of disease severity if the groin lesion had not been carefully examined. Contrast-enhanced CT proved crucial in demonstrating the femoral hernia, non-viable ileal segment and fistulous communication, and should be considered the imaging modality of choice when ECF is suspected in this setting.

Management of ECF is often summarized by the SNAP principle: control of Sepsis, optimization of Nutrition, definition of Anatomy and planning of a definitive Procedure.^10^ For femoral hernia-related ECF, urgent surgery is usually indicated because of ongoing strangulation. In the present case, early exploration allowed resection of the necrotic ileal segment and Meckel’s diverticulum with primary anastomosis after adequate lavage. Given the contaminated field, we avoided prosthetic mesh and performed primary tissue repair of the femoral defect, consistent with current recommendations.^1-3^ A diverting ileostomy was fashioned to protect the anastomosis, in line with strategies aimed at reducing leak-related morbidity.^10^

The outcome in our patient was favorable, with rapid recovery and no recurrence of fistula or hernia at short-term follow-up. This contrasts with reports where delayed diagnosis or inadequate nutritional and sepsis control led to prolonged hospitalization or mortality.^10^ Close follow-up is nonetheless required, as elderly patients may have limited physiological reserve and comorbidities.

This case has several limitations. It represents a single-patient experience, and the follow-up period is relatively short, precluding firm conclusions regarding long-term recurrence and quality of life. In addition, detailed nutritional and biochemical parameters were not systematically recorded.

Despite these limitations, the case underlines an important take-home message: in elderly women presenting with an irreducible groin swelling and atypical skin changes or feculent discharge, clinicians should maintain a high index of suspicion for femoral Richter’s hernia complicated by enterocutaneous fistula. Prompt CT imaging and early, tailored surgery can prevent life-threatening sepsis and improve outcomes.

## CONCLUSION

Spontaneous enterocutaneous fistula secondary to femoral Richter’s hernia is rare and may present with subtle abdominal signs. Careful examination of groin lesions, appropriate imaging and early surgery without mesh in contaminated fields are key to successful management in high-risk elderly population.
